# A Hybrid Spatio-Temporal Data Indexing Method for Trajectory Databases

**DOI:** 10.3390/s140712990

**Published:** 2014-07-21

**Authors:** Shengnan Ke, Jun Gong, Songnian Li, Qing Zhu, Xintao Liu, Yeting Zhang

**Affiliations:** 1 School of Software, Jiangxi Normal University, Nanchang 330022, China; E-Mail: consnan@126.com; 2 Department of Civil Engineering, Ryerson University, Toronto, Ontario M5B 2K3, Canada; E-Mails: snli@ryerson.ca (S.L.); xintao.liu@ryerson.ca (X.L.); 3 Faculty of Geosciences and Environmental Engineering, Southwest Jiaotong University, Chengdu 611756, China; E-Mail: zhuq66@263.net; 4 State Key Laboratory of Information Engineering in Surveying Mapping and Remote Sensing, Wuhan University, Wuhan 430079, China; E-Mail: zhangyeting@263.net

**Keywords:** trajectory, spatio-temporal data index, R-tree, B*-tree, cloud storage

## Abstract

In recent years, there has been tremendous growth in the field of indoor and outdoor positioning sensors continuously producing huge volumes of trajectory data that has been used in many fields such as location-based services or location intelligence. Trajectory data is massively increased and semantically complicated, which poses a great challenge on spatio-temporal data indexing. This paper proposes a spatio-temporal data indexing method, named HBSTR-tree, which is a hybrid index structure comprising spatio-temporal R-tree, B*-tree and Hash table. To improve the index generation efficiency, rather than directly inserting trajectory points, we group consecutive trajectory points as nodes according to their spatio-temporal semantics and then insert them into spatio-temporal R-tree as leaf nodes. Hash table is used to manage the latest leaf nodes to reduce the frequency of insertion. A new spatio-temporal interval criterion and a new node-choosing sub-algorithm are also proposed to optimize spatio-temporal R-tree structures. In addition, a B*-tree sub-index of leaf nodes is built to query the trajectories of targeted objects efficiently. Furthermore, a database storage scheme based on a NoSQL-type DBMS is also proposed for the purpose of cloud storage. Experimental results prove that HBSTR-tree outperforms TB*-tree in some aspects such as generation efficiency, query performance and query type.

## 1. Introduction

In recent years, there has been tremendous growth in the field of indoor and outdoor positioning sensors [1,2]. At the same time, precision and reliability in positioning have been enhanced dramatically, and mobile positioning services have become pervasive around the world. Users of positioning devices are not simply consumers of location information, and they also provide the content of geospatial information via Web 2.0 technologies. These phenomena result in unprecedented situations where databases are consistently populated with massive data—much of them are trajectory data. Further, dramatically developing techniques such as mobile computation, wireless transmission and ubiquitous positioning sensors continue to pose new challenges on spatio-temporal databases. Moreover, spatio-temporal search now concerns not only space but also time and trajectory semantics. In order to cope with these problems, spatio-temporal indexing techniques for trajectory data, namely trajectory indexing techniques, need to be studied as the core of data management solutions [3]. Moreover, the trajectory indexing technique deeply influences advanced spatio-temporal analyses, such as behavioral pattern analysis and intelligent transportation decision [4]. Yet, little progress has been made to date on creating generic tools for the analysis of trajectories [5].

Traditional indexing methods can be used to access trajectory data. For example, one-dimensional compound indexes based on B-tree variants can be built for multi-dimensional data such as spatio-temporal data, where multi-dimensional spatio-temporal data are transformed into one-dimensional sorting codes. However, spatio-temporal data aggregation deteriorates greatly in this way, which can lead to poor performance in spatio-temporal queries. Some spatial indexes such as R-tree and Octree can be easily extended into spatio-temporal indexes, in which time is viewed as another dimension in addition to spatial dimensions. However, if the spatial indexes are directly applied to time dimension, there will be some potential concerns over efficiency [6].

Existing trajectory indexes can be classified into three groups. The indexes in the first group are based on multi-version structures, in which each timestamp corresponds to a spatial index structure and unchanged nodes are shared between versions, such as HR-tree and MV3R-tree [7]. This type of index adopts a strategy which concerns firstly temporal dimension and secondly spatial dimensions. Its advantage is that its efficiency is high for a time slice query [8]. However, a new node in the index structures will be generated even if a node changes slightly, so the storage cost is high. Additionally, it leads easily to low efficiency in interval queries. The second type of index is based on spatial partition methods, such as SETI and CSE, in which trajectory points are firstly divided into respective spatial partitions and then a temporal index is generated for points in each partition [9]. Obviously, it applies a strategy which concerns firstly spatial dimensions and secondly temporal dimension. The main advantage of this category is that space is divided regularly so that generation and query efficiency are high [10]. However, the extent of space needs be predefined, and data distribution may be skewed. In addition, trajectories are explicitly split by the boundaries of divided partitions of predefined spatial extent. The third type of index, such as STR-tree and TB-tree, is usually extended from traditional spatial indexes such as R-tree, where temporal dimension plays the identical role as spatial dimension. TB-tree and its variants focus on explicit trajectory representation. This category can adaptively adjust index structures according to data distribution, which produces better query performance, but readily deteriorates index generation performance [11]. Very interestingly, a parallel indexing technique is proposed to transform spatio-temporal queries into parallel operations in different spatio-temporal dimensions [12].

Before a data index is studied and designed, it is essential to understand the data characteristics, e.g., its type and usage [6]. It is agreeable that there is not a universal indexing method that can satisfy requirements of all queries. Generally, indexing methods are influenced by a series of factors such as generation efficiency, storage utilization, query performance, query type and caching mechanism. Trajectory data typically exhibits the following properties: (a) update operations occur very frequently, (b) trajectory data volume tends to be huge, and (c) the types of trajectory queries are very diverse. Since moving objects change locations frequently, spatio-temporal data indexes also needs to be updated accordingly, which means particular emphasis should be put on the update efficiency. In the meantime, index data of only one million trajectory points surpass one hundred megabytes, and terabyte-level trajectory data sets are now common, so storage utilization has to be of concern. Moreover, adaptive caching mechanisms have to be adopted in indexing methods, which will avoid unnecessary resource occupation.

In order to synthesize the advantages of different indexes and make up for the existing methods, a hybrid indexing method has gradually become a trend [13]. Furthermore, cloud storage mechanism becomes a wise solution to trajectory databases, whose users are growing rapidly [14]. Focusing on the dynamic and semantic trajectory data organization over space and time, this paper presents a hybrid trajectory indexing method (HBSTR-tree), which combines Hash table, B*-tree and spatio-temporal R-tree. The remainder of the paper first introduces the design idea and the framework of the method in Section 2, followed by the detailed procedures and steps of the method in Section 3. Section 4 introduces a database scheme for storing indexes. Section 5 presents the experimental results and performance evaluation results in comparison with another outstanding method—TB*-tree [8]. Finally, some conclusions are given in Section 6.

## 2. Concept and Structure of HBSTR-Tree

**Figure 1.**
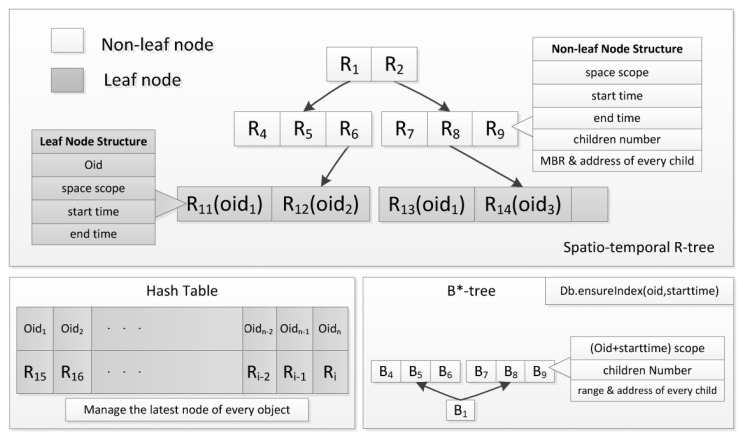
Framework of HBSTR-tree.

This section describes the concept and structure of the proposed index method, named HBSTR-tree, which supports real-time incremental trajectory databases. Figure 1 shows the principle and the framework of HBSTR-tree. The three sub-structures play different roles described as follows:

(a) Spatio-temporal R-tree is the principal part, which supports spatio-temporal range query;
(b) Hash table is the accessorial structure, which manages the latest nodes in cache before such nodes are inserted as leaf nodes into spatio-temporal R-tree in order to decrease insertion operations of spatio-temporal R-tree; and
(c) B*-tree is the secondary part, which is used as the temporal index for leaf nodes of spatio-temporal R-tree to query the trajectories of the targeted objects.

In trajectory databases, the attributes of a trajectory point includes object identity, time stamp and spatial coordinates, *etc.* Since the coordinates could be erroneous due to, for example, blocked GPS signals or unstable positioning sensors, some data cleaning processes are needed to filter these errors [15]. Usually a moving object may have hundreds of thousands of trajectory points itself if it is tracked for a long time. It is unwise to store them dispersedly, which will affect storage utilization and generation efficiency as well as query performance. Consecutive trajectory points are stored in a group as leaf nodes of spatio-temporal R-tree and a leaf node belongs to exactly one moving object. Practically, there may be a long time interval between adjacent trajectory points of an object. A time threshold called ***T****_t_* is defined. A new node will be created to store subsequent trajectory points when the time interval between two points surpasses this time threshold or when the number of trajectory points in the leaf node reaches the maximum capacity predefined by HBSTR-tree. It is helpful to avoid huge space-time coverage of leaf nodes.

The R-tree used in HBSTR-tree is a spatio-temporal one with ***N*** (2 or over) spatial dimensions and one temporal dimension. For such an R-tree, Minimum Bounding Rectangle (MBR) of one node is the minimum scope in spatio-temporal axes covering its children items. Here, the absolute number of seconds since 1 January 1970 00:00:00 UTC is used as the time reference, which Windows API and MongoDB API support. Unlike the original R-tree, leaf nodes are straightly inserted into the level-one nodes of spatio-temporal R-tree by a new insertion algorithm, where a novel node-choosing sub-algorithm is adopted. Hash table is used to manage the latest nodes of any moving object before these nodes are inserted as leaf nodes into R-tree, and these nodes can be accessed efficiently with object identification via Hash table. When a node becomes full, it will be inserted into spatio-temporal R-tree as one leaf node and a new node will be generated to substitute it. Following this strategy, trajectory points are inserted into R-tree in group, so the insertion operation will be more efficient.

The R-tree supports various query types, such as trajectories in spatio-temporal range, trajectories in spatial range and at time slice, and nearest neighbors over a time slice or a time range. However, it is ineffective to search for trajectories of targeted objects, which is commonly required in moving object database. In order to solve this problem, leaf nodes of spatio-temporal R-tree are indexed by a B*-tree, whose compound key comprises object identification (OID) and start time (StartTime) of leaf nodes. One-dimensional search capability of the B*-tree index helps to locate the leaf node covering trajectory points of a moving object at any time slice and then scan its trajectories via bidirectional pointers of B*-tree . Since a leaf node usually comprises almost 80 trajectory points, the B*-tree index of leaf nodes costs much less than the corresponding one of the trajectory points.

## 3. HBSTR-Tree Algorithm

Hash table and B*-tree are traditional indexing methods, and they are briefly introduced here with respect to how to use them in the proposed HBSTR-tree. Since spatio-temporal R-tree plays a more crucial role in HBSTR-tree as opposed to the former two, the procedures and steps of its algorithm will be described in detail in this section.

### 3.1. Spatio-Temporal Interval Criterion

Spatio-temporal interval criterion is defined to evaluate MBRs of nodes. The criterion is crucial for two sectors in R-tree generation, namely node-choosing and node-splitting sub-algorithms. For example, the original idea of node-choosing algorithm is to insert a tuple into the tree and the insertion will enlarge the involved nodes' MBRs as little as possible. Furthermore, if the insertion makes a node overflow, its child items will be split into two subsets whose MBRs cover as little as possible. In spatio-temporal R-tree, MBR is a spatio-temporal rectangle parallel to axes. According to original R-tree, the product of intervals in spatial and temporal axes, which is called spatio-temporal volume here, can be considered as a spatio-temporal interval criterion. According to this criterion, not cubic nodes but irregularly-shaped nodes may come first, which is prone to multi-path search, and this badly affects query performance. Seemingly, spatio-temporal volume roughly represents existence of moving objects. Actually, temporal dimension is very different from spatial dimensions for moving objects. Most moving objects repeat trajectories in space, but time in trajectory monotonically increases so that leaf nodes always expand in temporal dimension until full. Moreover, in common spatio-temporal search operations, regular shapes, such as square (2D) and cube (3D), are often used as spatial condition, and time condition can be moment or interval-based.

Therefore, in addition to time discrimination, shapes of MBRs should be similar to squares or cubes in space [16]. In view of this, a new spatio-temporal interval criterion is presented below.

Let the ranges in ***N*** spatial axes be (S_1_,S_2_,…,S_N_) and the range in one temporal axis be ***T***. Spatio-temporal interval criterion for ***N*** spatial dimensions and one temporal dimension is defined as follows:

(1) $$C={\left(\frac{\sum_{N}^{i=1}{\text{S}}_{i}}{N}\right)}^{N}\times T$$

The reason as to why to adopt it originates from the Inequality of Arithmetic and Geometric Means, briefly the AM-GM inequality. Below is our explanation of this criterion.

For three positive quantities ***X***, ***Y***, and ***Z***:

(2) $$\begin{array}{cc} {\left(\frac{X+Y+Z}{3}\right)}^{3}\geq \text{X}*\text{Y}*\text{Z}, & \;\text{with equality if and only if X = Y = Z} \end{array}$$

The left part of the inequality is the Arithmetic Means of ***X***, ***Y*** and ***Z***, and its right part is the Geometric Means of ***X***, ***Y*** and ***Z***. Given a fixed volume, there is the minimum Arithmetic Means only when it is a cube, whose sides are equally long. This can be generalized into N-dimensional space. The less the Arithmetic Means of ***N*** spatial sides of MBR are, the more likely the ranges of the spatial axes of MBR are equal. Since the Arithmetic Means of ***N*** spatial axes of MBR are introduced as part of the spatio-temporal interval criterion, this criterion will be helpful to control node shapes. Furthermore, if ***T*** is less, the criterion will be less. According to this criterion, MBR can be evaluated and optimized for spatio-temporal queries.

### 3.2. HBSTR-tree Insertion Algorithm

In order to improve network transmission, moving objects usually accumulate some trajectory points locally and then collectively upload them to data servers. In addition, moving objects are sometimes offline. As a result, trajectory points are temporally sequential for one object, but it cannot be ensured that trajectory points from all objects are consecutive in time. This factor is taken into account in the HBSTR-tree algorithm. The HBSTR-tree involves some basic indexing structures, such as R-tree, B*-tree and Hash table. These structures are interrelated and interact with each other. Like other R-tree variants, HBSTR-tree is built by inserting trajectory points one by one, so its generation algorithm is called an insertion one, which includes some key sub-processes, such as node-choosing and node-splitting.

In order to satisfy secondary storage, every node in R-tree corresponds to one disk page, whose size depends on basic parameters of operating systems and database and can be set according to hardware and software context. At the same time, page size decides the number of child items in a node. The structure of leaf nodes is different from that of non-leaf nodes. Therefore, there are different maximum numbers of entries or child items in leaf nodes and non-leaf nodes. In order to ensure that the level number of every node is unchanged in its lifecycle, let the level number of leaf nodes be 0, and from bottom-up the level number of nodes in the other levels is sequentially plus one. For example, the father of a leaf-node is a level-1 node. According to R-tree generation mechanism, the depth of R-tree will not change until the root node is split into two small nodes. Then, one new root node is generated and becomes the father of the two nodes. Hence, the new root node's level number is the level number of the old root node plus one.

The following describes the detailed steps of the HBSTR-tree insertion algorithm. The Node-splitting sub-algorithm is similar to the original one, but the node-choosing sub-algorithm is substantially different from the original one. Therefore, this paper focuses on the node-choosing sub-algorithm. The spatio-temporal interval criterion mentioned above is adopted in both of the sub-algorithms.

**Figure 2.**
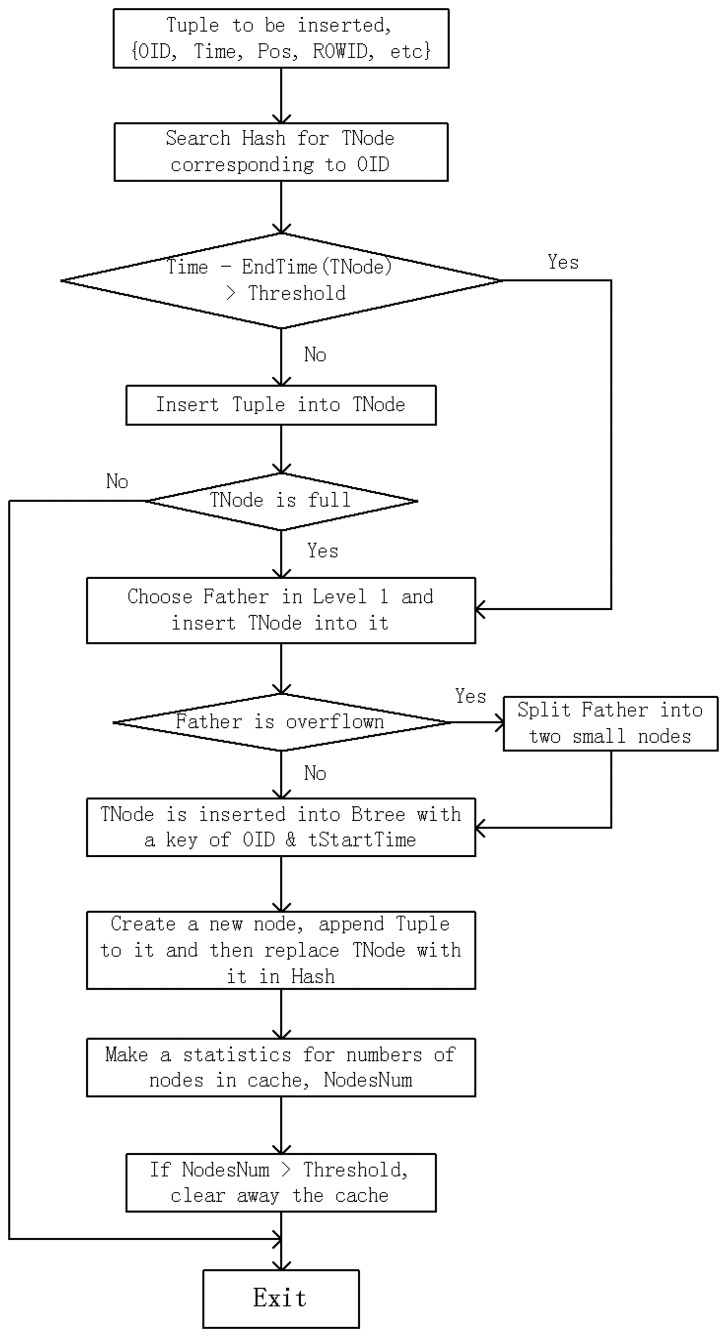
Flowchart of HBSTR-tree insertion algorithm.

Figure 2 illustrates a simplified flowchart of this algorithm. The trajectory point to be inserted into index is Tuple{OID, Time, Pos, ROWID, *etc.*}, in which OID is object identity code, Time is the timestamp, Pos is the location of trajectory point, and ROWID is the pointer or storage address of trajectory point record. HBSTR-tree includes Hash table named Hash (OID mapped to the latest leaf node of moving objects), B*-tree named Btree (whose key is OID/start time of the leaf node), and spatio-temporal R-tree named Rtree. Then this algorithm is described in pseudo code in Algorithm 1.

**Algorithm 1.** Pseudo code of HBSTR-Tree insertion algorithm.
**Algorithm 1 Description: HBSTR-Tree Insertion Algorithm**
**Input: Tuple** (inserted trajectory point), **HBSTR** (existing index structure), **T_thres_** (time threshold for continuous trajectory), **Tcnode** (time threshold for cache clearance)**Output**: updated index
1. Search in Hash for the node corresponding to OID, **TNode**.2. **If Time**–EndTime (**TNode**) < **T_thres_ Then**3. Insert **Tuple** into **TNode**4. **If** TNode is not full **Then**5. **Exit**6. **End If**7. **End If**8. Use **node-choosing sub-algorithm (Algorithm 2)** to pick out the most suitable node in Level 1 of **Rtree, Father,** as the father of **TNode** and then insert **TNode** into **Father**9. **If** Father is overflown **Then**10. Use node-splitting sub-algorithm to divide child items of Father into two small nodes. This operation may lead to the overflow of upper-level nodes, and then recursively handle it even till root node11. **End If**12. OID and StartTime, which are the attributes of **TNode**, are combined as an index key OID/StartTime and inserted into **Btree** as an index entry13. Create a new leaf node, and append **Tuple** to it. Then replace **TNode** with this new leaf node in **Hash**14. Make a statistics for the number of nodes in cache, called **NodesNum**. If **NodesNum** surpasses the threshold, clear away the nodes in cache which have not been accessed for **T_cnode_**


HBSTR-tree uses Hash to find the leaf node, in which the tuple should be inserted. Only when the leaf node is full after insertion, the leaf node will be inserted into R-tree. Therefore, the worst case of the algorithm is the latter case. If node-choosing and node-splitting are atomic operations, the worst-case complexity of insertion algorithm is O(logN) because the tree depth is O(logN). The best case is that leaf node is not full and the tuple is straightly inserted into leaf node. The complexity in this case is O(1) because the main cost is to find the leaf node with Hash. Fortunately, the probability of the best case is M times greater than that of the worst case, in which M is the fanout-maximum capacity of leaf nodes.

### 3.3. Node-Choosing Sub-Algorithm

The node-choosing sub-algorithm in the HBSTR-tree is different from traditional ones. Firstly, it searches for the lowest-level nodes that completely contain the leaf node to be inserted into R-tree. Such lowest-level nodes may be in any level of R-tree or do not exist. Obviously, the child nodes of these lowest-level nodes will not contain the leaf node. Then, one node is selected from these child nodes, which will change the least after insertion of the leaf node. Finally, assuming that the selected node is the root node, the level-one node will be picked out in a top-to-bottom way by the original node-choosing procedure. This algorithm can avoid the erroneous result caused by node overlap. Algorithm 2 illustrates the node-choosing sub-algorithm procedure in pseudo code, which is implemented in non-recursive mode. Although this algorithm looks more complex than the recursive one, it is actually more efficient. Its time complexity is O(logN) corresponding to the tree depth. Although it may probe multiple paths to choose the proper node, it can help to generate smaller node coverage and better tree shape.

**Algorithm 2.** Pseudo code of node-choosing sub-algorithm.
**Algorithm 2 Description: node-choosing sub-algorithm** (pick out one node at Level 1, into which TNode will be inserted)
**Input: TNode** (leaf node to insert into R-tree), **Root** (the root of R-tree)**Output:** a level-one node (considered as the father of TNode)
1. NodeSet.Clear()            : temporal node set is cleared off2. MinLevelID → **Root**.LevelID + 1     : let MinLevelID be tree depth initially3. Father → **Root**           : let Father be Root4. SeqArray[Father.LevelID] → 0;      : let the Father.LevelID^th^ item in SeqArray be 05. **While** Father != NULL **Do**6. Node → Father.IthChild (SeqArray[Father.LevelID])     : let the i^th^ child of Father be the current node7. **If** Node.Contain(**TNode**) = True **Then**     : if Node contains TNode8. **If** Node.LevelID = MinLevelID **Then**     : if level number of Node is equal to MinLevelID9. NodeSet.Add(Node)     : add Node in NodeSet10. **Else If** Node.LevelID < MinLevelID **Then**     : if level number of Node is less than MinLevelID11. NodeSet.Clear()     : clear NodeSet12. NodeSet.Add(Node)     : add Node in NodeSet13. MinLevelID → Node.LevelID     : let MinLevelID be level number of Node14. **End If**15. **If** Node.LevelID > 1 **Then**     : if level number of Node is greater than 1, enter the lower level16. Father → Node     : let Father be Node17. SeqArray[Father.LevelID] → 0     : let the corresponding item in SeqArray 018. **Continue Loop**     : restart new loop19. **End If**20. **End If**21. **If** Node.LevelID = 1 Or Node.Contain(**TNode**) = False **Then**     : if Node is at level 1 or Node doesn't contain Leaf22. SeqArray[Father.LevelID] → SeqArray[Father.LevelID] + 1     : move to next child node23. **While** SeqArray[Father.LevelID] = Father.NumChildren **Do**     : if finish traversing all child nodes24. Father → Father.ParentNode     : backspace to upper level25. **If** Father = NULL **Then**     : if finish traversing all child nodes of Root26. **Break Loop**     : exit loop27. **End If**28. SeqArray[Father.LevelID] → SeqArray[Father.LevelID] + 1     : start traversing the right sibling29. **End While**30. **End If**31. **End While**32. **If** NodeSet.IsEmpty() = True **Then**     : if NodeSet is null, there is no node to contain TNode33. NewRoot → **Root**     : let NewRoot be Root34. **Else If** MinLevelID = 1 **Then**     : if there exist the required nodes at level 135. NewRoot → LargestNode(NodeSet)     : let NewRoot be the largest node in NodeSet36. **Else**37. Set is the collection of child nodes of all nodes in NodeSet. In Set, choose a node which will change the least after containing TNode. Let NewRoot be the node38. **End If**39. In the sub-tree whose root is NewRoot, search for a node in level 1 by classical node choosing algorithm.40. Output the node. **Exit**


## 4. Storage Scheme for Spatio-Temporal R-Tree Index

In consideration of huge data volume, MongoDB is readily adopted as the storage tool for trajectory data and HBSTR-tree. MongoDB is a NoSQL-type DBMS, which has some capabilities key to Web 2.0 applications, such as cloud storage, good read/write performance and scheme-free. The elements in the structure of MongoDB include database, dataset, document and element. A dataset corresponds to a table in a traditional database. Documents are similar to records, but they are not required to have the fixed structures in one dataset. Hence, it can easily store data contents with varied structure, such as R-tree nodes. Elements are similar to fields.

This section focuses on the storage scheme of spatio-temporal R-tree in MongoDB. The R-tree index is stored in a dataset. In order to be recognized easily, the name of the spatio-temporal index dataset is labeled by the name of a corresponding trajectory dataset with “.STI_Index” as a suffix. The metadata of the R-tree index includes some basic information, such as database name, dataset name, index dataset name, spatial dimensions, fan-out parameters of R-tree, node numbers, and root node's ROWID. Root node's ROWID is the only identity of root node document. Only via it, root node can be found in a database, and then the whole tree can be further visited. Every document in MongoDB has ROWID, which can be user-designated or allocated autonomously according to server, process and time, *etc.* It is an array of 12 characters, which is essential for MongoDB distributing storage.

In order to conveniently search for the metadata, metadata is stored together in one document whose ROWID is user-designated as “999999999999”. Besides metadata, every R-tree node is also a document in dataset. Nodes are classified into two types, leaf node and non-leaf node. Leaf node comprises consecutive trajectory points. Non-leaf node includes the entries of all its child nodes. For convenience's sake, both node data and metadata are respectively organized into binary data block called BinData type as document elements. Given below is the storage scheme of spatio-temporal R-tree in HBSTR-tree as references. Table 1 introduces the document structure of the metadata of spatio-temporal R-tree, which has only one metadata document in the R-tree. Table 2 lists the elements of MetaInfoData in the metadata document, and MetaInfoData is a binary data block. Table 3 describes the document structure of R-tree node. One node, no matter whether or not it is a leaf node, corresponds to one document. Table 4 describes NodeBufData, which is an element of node document, and introduces the differences between leaf node and non-leaf node.

## 5. Experimental Studies

In this section, the technical aspects of the HBSTR-tree are tested, such as index generation and query process, and the experimental results are compared with those of TB*-tree. TB*-tree is chosen due to its superior performance in trajectory query [8]. TB*-tree originates from TB-tree and keeps its basic advantages in trajectory preservation. TB*-tree uses Guttman's original algorithm to delete and reinsert the leaf nodes which are full. TB*-tree concerns both space dimensions and time dimensions in the same way, not like TB-tree which first focuses on time. Leaf nodes of TB*-tree belonging to the same object are connected with a double-linked list chronologically, which greatly helps searching for the trajectory efficiently and effectively. TB*-tree is optimized in this paper for fair comparison, and its leaf nodes will only be inserted into R-tree if HBSTR-tree is full, which will be more efficient than the original TB*-tree.

### 5.1. Experimental Setup

The page size for spatio-temporal R-tree in HBSTR-tree is chosen to be 3 KB resulting in a fanout maximum capacity of 80 and 40 for the leaf and non-leaf nodes, respectively. The fanout minimum capacity for non-leaf nodes is 16, which is 40% of the maximum capacity. Because TB*-tree structure is almost the same as spatio-temporal R-tree in HBSTR-tree except the double-linked pointers in leaf nodes in TB*-tree, their fanout parameters can be set as the same. Double-type data (64-bit float) is used for Space coordinates and Time-type data (64-bit integer) for time coordinates. The experiments were done in a computer with 64-bit Window 7 Operating system, MongoDB (64-bits), CPU Intel Core I7-3770M 3.40 G, 8 GB RAM, and 1 TB hard disk (7200 rpm, 64MB cache).

In order to achieve scalability in the cardinality of the datasets and study the behavior of the index structures under several settings, we used the Brinkhoff's spatio-temporal data generator, which are widely used to generate synthetic datasets for benchmarking spatio-temporal database [17]. The details of the experimental datasets are given in Table 5. The four datasets in different scales are generated based on the road network data of City of Oldenburg, Germany, and they exist in the same spatio-temporal scope. The four datasets cover the same time and space range, and include the different scales of objects and trajectory points.

### 5.2. Time Performance and Storage Efficiency of Index Generation

Trajectory databases are commonly of huge volume, so index generation performance is a very crucial aspect. A comparative experiment is made between TB*-tree and HBSTR-tree. The quadratic split strategy in original R-tree is adopted in these two trees. Table 6 is the experimental results for index generation, including time cost and storage cost. Take Dataset O5000K as example. TB*-tree costs 439 s to build index whereas HBSTR-tree costs 454 s. Here, time costs cover the time taken to access original data and store index data.

Assuming that ***N*** is the number of entries (trajectory points) and ***M*** is the fanout maximum capacity of leaf nodes, time complexity of both TB*-tree and HBSTR-tree generation is ***O(N/M)*** if the insertion operation is considered as an atomic operation. R-tree insertion operation is time-costing which includes node-choosing and node-splitting. The node-choosing operation in HBSTR-tree is a little more complex than TB*-tree, and their time complexities are ***O(log_M_ N)***. Except for spatio-temporal interval criterion, their node-splitting procedures are identical to the original R-tree, so the time complexity is ***O(L****^2^****)*** (assuming that ***L*** is the fanout maximum capacity of non-leaf nodes). The number of node-choosing and node-splitting in the process of building HBSTR-tree is a little greater than TB*-tree, so the time cost of HBSTR-tree is higher. Interestingly, the number of node-choosing operations is equal to the number of leaf nodes, and the number of node-splitting operations is equal to the number of non-leaf nodes.

An often neglected aspect in benchmarking access methods is data size of index structures. HBSTR-tree has one in-memory sub-index (Hash) and two in-disk sub-indexes (R-tree and B*-tree), so its storage cost is the sum of R-tree and B*-tree. For Dataset O5000K, the data size of TB*-tree is 217 MB, whereas that of HBSTR-tree is 221 MB where the sizes of two sub-indexes are 216 MB and 5 MB, respectively. Because the fanout minimum capacity in non-leaf nodes is 16, the majority of those nodes in R-tree, over 94%, are leaf nodes. Most leaf-node pages in HBSTR-tree can be fully utilized because of the HBSTR-tree mechanism. In HBSTR-tree, the compound B*-tree only indexes leaf nodes of R-tree, and the index key is OID/timestamp of leaf nodes, so its storage cost is subtle as opposed to R-tree. For trajectory preservation, the B*-tree keeps the double-linked list among leaf nodes, which need extra space. As opposed to the original dataset whose size is 675 MB, both the TB*-tree and HBSTR-tree are compressed greatly.

### 5.3. Spatio-Temporal Range Query Process

The most common operation in trajectory database is to search for trajectory points in spatio-temporal ranges. This section presents performance comparisons and analyses about range queries between TB*-tree and HBSTR-tree. We use the following set of three range queries (Q1–Q3): three sets of 100 random query windows with 1%, 2% and 4% of every axial range of the valid space, respectively, over the synthetic data increasing the number of trajectory points and moving objects (O5000K–O40000K datasets).

**Figure 3.**
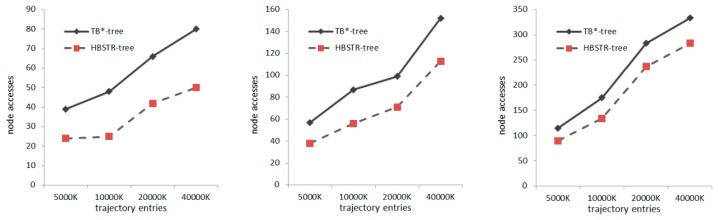
Spatio-temporal range queries (**left**) Q1(1%); (**middle**) Q2(2%); (**right**) Q3(4%).

The average of 100 window queries is considered as the test result. The experimental results of range queries in different scales are illustrated in Figure 3. Obviously, HBSTR-tree has superior range query performance over its competitor regarding the queries with sizes of 1%(Q1), 2%(Q2) and 4%(Q3) of every valid axial range. TB*-tree and HBSTR-tree have the same set of leaf nodes, and the leaf nodes in both trees hit by range query are identical too. When the query range is greater, the leaf nodes will be more easily hit and cover greater percentage of the total accessed nodes. Meanwhile, leaf nodes are far more than non-leaf nodes. As a result, when the query range becomes greater, the absolute difference in the number of accessed nodes will still increase but the relative difference will decrease.

### 5.4. Trajectory Query for Targeted Object

The most common operation is to find trajectories of targeted objects in a period of time. One hundred moving objects are randomly selected from the four datasets, and then 10% of their life cycle is randomly selected as the query conditions in our experiments about trajectory query. Table 7 illustrates the experimental results over those four datasets. The average of node accesses in these 100 trajectory query operations is summed up.

In HBSTR-tree, B*-tree sub-tree directly locates the first leaf node which satisfies query condition, then scan backwards and output the required leaf nodes one by one, so that redundant node accesses are avoided. On the contrary, TB*-tree searches the tree structure from the root node. After the first targeted leaf node is hit, the double-linked list between leaf nodes will be used to find the satisfying leaf nodes. Obviously, this search procedure is not stable and sometimes may traverse the whole tree. Since 10% of consecutive trajectory points of an object exist in one or two leaf nodes, the HBSTR-tree only needs to access one or two leaf nodes. In addition to this, B*-tree sub-index need be accessed. The node access of B*-tree should be also concerned in evaluating trajectory queries. If the maximum number of keys in a B*-tree node is 100, the level number of B*-tree for O5000K, O10000K, O20000K and O40000K is 3. During a search operation, one node in each level of B*-tree is accessed.

## 6. Conclusions

A trajectory database is usually of a large volume, and the data distribution is skewed in space-time. Meanwhile, real-time response speed and rich semantics are urgently needed for trajectory queries in nowadays' applications. This paper presents a novel trajectory indexing method, which synthesizes the advantages of R-tree, B*-tree and Hash table. A new spatio-temporal interval criterion and a novel node-choosing algorithm are proposed to improve the spatio-temporal R-tree sub-index. This index has good generation efficiency, and outperforms TB*-tree in several aspects, such as range query and targeted trajectory query. Meanwhile, it satisfies real-time update and supports common trajectory query types. Moreover, this paper proposes a practical database scheme based on MongoDB, which belongs to NoSQL DBMS and supports cloud storage.

Large-scale, real-time trajectory data management is a key technique in intelligent transportation systems and social network services. The further work will focus on process simulation and analysis for complex geoscience problems, which benefits urban management and policy decisions.

## Figures and Tables

**Table 1.** Structure of index metadata document.

**Element**	**Type**	**Description**
_id	ROWID	Identity of metadata document (999999999999)
MetaInfoData	BinData	Includes a series of metadata

**Table 2.** Description of MetaInfoData element.

**Element**	**Type**	**Description**
DBName	string	Name of DB which includes original dataset(for example, DBNAME.DATASETNAME)
DatasetName	string	Name of original dataset corresponding to Index(for example, DBNAME.DATASETNAME)
IndexDatasetName	string	Name of index dataset
SpatioDimensions	Int	Spatial dimensions
MaxNodesNum	Int	Maximum of fanout parameters in non-leaf node
MinNodesNum	Int	Minimum of fanout parameters in non-leaf node
MaxTuplesNum	Int	Maximum of fanout parameters in leaf node
MinTuplesNum	Int	Minimum of fanout parameters in leaf node
TotalNodesNum	Int	Number of the total nodes
RidTreeRootNode	ROWID	ROWID of root node

**Table 3.** Description of document structure for R-tree node.

**Element**	**Type**	**Description**
_id	ROWID	Unique ROWID of node
NodeBufData	BinData	Node data in BinData type

**Table 4.** Description of element structure for NodeBufData.

**Element**	**Type**	**Description**
LevelID	Int	Level number(leaf node belongs to Level 0)
Interval	SpatioTemporalInterval	MBR of this node
ridParentNode	ROWID	ROWID of father node
NumChildren	Int	Number of child entries
RidChildren	Array(ROWID)	If leaf node, here are ROWIDs of trajectory points; if non-leaf node, here are ROWIDs of child nodes

*On the condition of leaf node*		

ObjectID	*ObjectIDType*	*Object identity*
ChildPoints	*Array(SpatioTemporalPoint)*	*Trajectory point array, whose size is NumChildren*

*On the condition of non-leaf node*		

ChildIntervals	*Arrary(SpatioTemporalInterval)*	*MBRs of child nodes, whose size is NumChildren*

**Table 5.** Description of experimental datasets.

**Name**	**# Time Stamps**	**x_min_, y_min_, x_max_, y_max_**	**# Objects**	**# Entries**
O5000K	1000	281, 3935, 23854, 30851	14000	5271991
O10000K	1000	281, 3935, 23854, 30851	26000	10579838
O20000K	1000	281, 3935, 23854, 30851	48000	20845388
O40000K	1000	281, 3935, 23854, 30851	90000	40819855

**Table 6.** Experimental results of index generation (Dataset O5000K).

**Index**	**Time Cost (s)**	**# Node Pages**	**# Node Choosing**	**# Node Splitting**	**Storage Costs (M)**
TB*-tree	439	76652	73734	2918	217
HBSTR-tree	454	76814	73734	3080	221 (216 + 5)

**Table 7.** Node accesses in trajectory queries.

**Index Type**	**Node Accesses**			

	O5000K	O10000K	O20000K	O40000K
TB*-tree	52	77	122	178
**HBSTR-tree**	5(2+3)	5(2+3)	5(2+3)	5(2+3)
